# Oral health in patients with different sites of head and neck squamous cell carcinoma is not different

**DOI:** 10.1371/journal.pone.0293665

**Published:** 2023-10-26

**Authors:** Philipp Kanzow, Katharina Mielke, Franziska Haupt, Susanne Wiegand, Henning Schliephake, Dirk Beutner, Annette Wiegand

**Affiliations:** 1 Department of Preventive Dentistry, Periodontology and Cariology, University Medical Center Göttingen, Göttingen, Germany; 2 Department of Otolaryngology, Head and Neck Surgery, University of Leipzig, Leipzig, Germany; 3 Department of Oral and Maxillofacial Surgery, University Medical Center Göttingen, Göttingen, Germany; 4 Department of Otorhinolaryngology, Head and Neck Surgery, University Medical Center Göttingen, Göttingen, Germany; Justus Liebig University Giessen, GERMANY

## Abstract

Oral health might not only act as risk factor for head and neck squamous cell carcinoma (HNSCC), but might also have a predictive value for the patients’ survival. Currently, information on the effect of oral health on survival of patients with different sites of HNSCC is lacking. This single-center retrospective study aimed to compare oral health in patients with different sites of HNSCC and to analyse whether oral health is associated with survival in the different subsets of HNSCC patients. Dental records of HNSCC patients referred for dental assessment prior to radio(chemo)therapy were included. Patient-related parameters (age at time of diagnosis, sex, tobacco exposure, alcohol consumption, HPV status), treatment data (primary treatment, intent), performance status, tumor demographics (anatomical site, TNM staging), and oral health parameters (DMFT, periodontal health, teeth with/without root canal treatment and with/without periodontitis apicalis) were obtained. Oral health parameters were compared between different anatomical sites. Survival of all HNSCC patients and of individual subsets was assessed using Kaplan-Meier statistics, and the effect of tumor demographics, patient-related parameters, and oral health on survival was analysed by cox regression analyses (α = 5%). 371 patients with HNSCC (oral: n = 86, oropharyngeal: n = 174, hypopharyngeal: n = 59, laryngeal: n = 15, other: n = 37) were included. Oral health parameters did not differ between subsets (p_adj._≥0.199). Five-year cumulative survival of HNSCC patients amounted to 78.6%. Only for HNSCC originating in the oral cavity and oropharynx, survival was associated with the treatment intent (p = 0.015) or performance status (p = 0.007) in the multivariable analyses, respectively. Within the limitations of this study, oral health was not different between different subsets and had no significant effect on survival of HNSCC patients.

## Introduction

Patients with head and neck squamous cell carcinoma (HNSCC) often present a poor oral health already at the time of diagnosis [1,2], which is then further reduced during radio(chemo)therapy [3,4]. Besides classical risk factors, such as tobacco exposure, alcohol abuse, or human papillomavirus (HPV) infection, several previous studies found poor oral hygiene or poor oral health status to be independent risk factors for HNSCC [5–9]. In these studies, squamous cell carcinomas that originate in the oral cavity, oropharynx, hypopharynx, or larynx were grouped as the same disease, although the anatomical site, prognostic markers (e.g. HPV-infection), treatment paths, and survival might be different among the subsets. Interestingly, oral health in different subsites of HNSCC was rarely investigated, and studies found conflicting results: Rupe et al. [1] found no difference in the number of decayed, missing, filled teeth (DMFT) and periodontitis between different sites, while Patel et al. [2] found significant differences with regard to DMFT, the number of present teeth, and horizontal bone loss. Tasoulas et al. [10] also found significant differences in the number of missing teeth.

Consequently, very little information on a possible association between oral health and different sites of HNSCC is available. Moreover, oral health might not only act as risk factor for HNSCC or HNSCC subsets, but might also have a predictive value for the patients’ survival: Interview-reported oral health parameters (e.g. regular dental visits, daily cleaning of teeth, use of dental floss) showed an association with or had an significant effect on survival of patients with different sites of HNSCC (i.e. oral, oropharyngeal, hypopharyngeal, laryngeal) [10–12]. Two of these studies also compared different HNSCC subsets and found varying effects of oral health parameters on survival across HNSCC subsets [10,12]. However, information on the effect of objective oral health data (dental and periodontal status) on survival of patients with different sites of HNSCC is lacking. Only for patients with oropharyngeal squamous cell carcinoma, the effect of periodontal scores obtained from orthopantograms on survival was assessed. Despite an association with survival, periodontal scores did not predict survival in a multivariable analysis [13].

Thus, this study aimed to compare oral health in different anatomical sites of HNSCC and to analyse whether objective oral health data is associated with survival in the different subsets. The null hypothesis is that oral health parameters are not significantly different between different anatomical sites of HNSCC patients.

## Materials and methods

This retrospective study was approved by the Ethics committee of the University Medical Center Göttingen (17/10/19). Patients were included from a single-center (University Medical Center Göttingen) which is a maximum care medical center. Potentially eligible patients were automatically derived from electronic health records from the Department of Oral and Maxillofacial Surgery and the Department of Otorhinolaryngology, Head and Neck Surgery based on pre-defined ICD-10 codes (C00-C14, C44.0-C44.4, C44.9, D37.0, D37.9). Afterwards, electronic and paper-based health records of identified patients were manually screened between May 18th 2020 and February 10th 2023. Patients with HNSCC, that were planned for radio(chemo)therapy and referred for dental assessment at the Department of Preventive Dentistry, Periodontology and Cariology between 2009 and 2019 were included. The dataset was anonymized prior to the statistical analysis.

The following patient-related parameters were obtained from electronic and paper-based patient files: anatomical site (oral, oropharyngeal, hypopharyngeal, laryngeal, other), age at time of diagnosis, sex, tobacco exposure (never smoker vs. former / current smoker), alcohol consumption (no vs. previously / currently), HPV status (HPV-negative vs. HPV-positive), primary treatment (surgery +/- adjuvant radiotherapy vs. radiotherapy / radiochemotherapy), treatment intent (curative vs. palliative), performance status (Karnofsky scale), and tumor/node/metastasis (TNM) staging.

Oral health data included dental status and data on periodontal health. For assessment of dental status, the number of present teeth and the DMFT score (decayed, missing, filled teeth) were obtained from patient files and validated using intra-oral or panoramic radiographs. The number of teeth with and without root canal treatment, the number of teeth with or without periodontitis apicalis and data on periodontal health were derived from intra-oral or panoramic radiographs [14,15]. For each patient, the averaged radiographic bone loss across all teeth was measured, expressed as absolute (mm) and relative (%) values as well as a function of age (%/age) [16]. The presence of subgingival calculus and radiographic furcation involvement was also assessed. All radiographs were evaluated by a calibrated examiner (KM).

### Statistical analysis

Oral health parameters were compared between different anatomical sites of HNSCC patients using Fisher’s exact test (dichotomous variables) or Kruskal-Wallis test (ordinal or continuous variables). Resulting p-values were adjusted for multiple testing according to Benjamini & Hochberg.

Survival was assessed using Kaplan-Meier statistics separately for all patients (i.e. all anatomical sites) and for each individual subset and compared by log-rank test. For each patient, the survival time was calculated from the date of diagnosis until event (death) or last known visit to the University Medical Center Göttingen (censoring). Separately for all patients and for each subset, the effect of patient-related risk factors (i.e. age at diagnosis, sex, tobacco exposure, alcohol consumption, HPV status), primary treatment, treatment intent, TNM staging, and oral health parameters was first analysed by univariable cox regression analyses and likelihood ratio tests (α = 10%). Subsequently, variables with a p-value ≤0.1 within the univariable analyses were entered in multivariable cox regression analyses (α = 5%).

All analyses were performed using the software R (version 4.2.1; www.r-project.org). For the time-to-event analyses, the packages “survminer” (version 0.4.9) and “survival” (version 3.5-0) were used.

## Results

Three hundred and seventy-one patients with HNSCC (oral: n = 86, oropharyngeal: n = 174, hypopharyngeal: n = 59, laryngeal: n = 15, other: n = 37) were included in this study. The median follow-up time amounted to 1.7 years (min: 0.0, max: 12.3 years, IQR: 0.6–4.3 years). Full tumor demographics, patient-related parameters, primary treatment, treatment intent, TNM staging, and oral health status for all HNSCC patients and separately for each subset are shown in Table 1. None of the assessed oral health parameters varied between subsets of different anatomical sites (p_adj._≥0.199).

**Table 1:** Univariate comparison of tumor demographics, patient-related parameters, and oral health status for each subset.

		Total (n = 371)	Oral (n = 86)	Oropharyngeal ^a^ (n = 174)	Hypopharyngeal (n = 59)	Laryngeal (n = 15)	Other (n = 37)
Age at diagnosis, mean±SD		64.7 ± 11.5	62.7 ± 12.5	63.8 ± 10.6	65.5 ± 8.9	66.4 ± 7.8	71.8 ± 15.5
Sex, n (%)	female	90 (24.3)	25 (29.1)	46 (26.4)	10 (16.9)	0 (0.0)	9 (24.3)
	male	281 (75.7)	61 (70.9)	128 (73.6)	49 (83.1)	15 (100.0)	28 (75.7)
Tobacco exposure, n (%)	never smoker	173 (46.6)	39 (45.3)	78 (44.8)	24 (40.7)	4 (26.7)	28 (75.7)
	former / current smoker	198 (53.4)	47 (54.7)	96 (55.2)	35 (59.3)	11 (73.3)	9 (24.3)
Alcohol consumption, n (%)	never	234 (63.1)	50 (58.1)	117 (67.2)	31 (52.5)	7 (46.7)	29 (78.4)
	former / current	137 (36.9)	36 (41.9)	57 (32.8)	28 (47.5)	8 (53.3)	8 (21.6)
HPV status, n (%) *(n = 185)*	HPV-negative	108 (29.1)	21 (24.4)	50 (28.7)	27 (45.8)	7 (46.7)	3 (8.1)
	HPV-positive	77 (20.8)	2 (2.3)	69 (39.7)	4 (6.8)	1 (6.7)	1 (2.7)
Primary treatment, n (%) *(n = 370)*	surgery +/- adj. radiotherapy	322 (87.0)	82 (95.3)	149 (85.6)	47 (81.0)	13 (86.7)	31 (83.8)
	radiotherapy / radiochemotherapy	48 (13.0)	4 (4.7)	25 (14.4)	11 (19.0)	2 (13.3)	6 (16.2)
Treatment intent, n (%)	curative	342 (92.2)	79 (91.9)	163 (93.7)	50 (84.7)	13 (86.7)	37 (100.0)
	palliative	29 (7.8)	7 (8.1)	11 (6.3)	9 (15.3)	2 (13.3)	0 (0.0)
Performance status, mean±SD *(n = 331)*		85.4 ± 13.2	84.1 ± 15.1	87.7 ± 11.7	82.5 ± 14.0	83.8 ± 17.1	83.3 ± 10.8
T stage, n (%)	x	9 (2.4)	0 (0.0)	3 (1.7)	0 (0.0)	0 (0.0)	6 (16.2)
	T0	1 (0.3)	0 (0.0)	0 (0.0)	0 (0.0)	0 (0.0)	1 (2.7)
	T1	72 (19.4)	25 (29.1)	33 (19.0)	5 (8.5)	1 (6.7)	8 (21.6)
	T2	117 (31.5)	28 (32.6)	57 (32.8)	21 (35.6)	4 (26.7)	7 (18.9)
	T3	91 (24.5)	20 (23.3)	49 (28.2)	11 (18.6)	2 (13.3)	9 (24.3)
	T4	81 (21.8)	13 (15.1)	32 (18.4)	22 (37.3)	8 (53.3)	6 (16.2)
N stage, n (%)	x	4 (1.1)	2 (2.3)	1 (0.6)	0 (0.0)	0 (0.0)	1 (2.7)
	N0	104 (28.0)	40 (46.5)	27 (15.5)	14 (23.7)	4 (26.7)	19 (51.4)
	N1	55 (14.8)	11 (12.8)	30 (17.2)	8 (13.6)	3 (20.0)	3 (8.1)
	N2	176 (47.4)	29 (33.7)	105 (60.3)	24 (40.7)	7 (46.7)	11 (29.7)
	N3	32 (8.6)	4 (4.7)	11 (6.3)	13 (22.0)	1 (6.7)	3 (8.1)
M stage, n (%)	x	43 (11.6)	17 (19.8)	14 (8.0)	2 (3.4)	0 (0.0)	10 (27.0)
	M0	306 (82.5)	66 (76.7)	156 (89.7)	50 (84.7)	12 (80.0)	22 (59.5)
	M1	21 (5.7)	3 (3.5)	4 (2.3)	7 (11.9)	3 (20.0)	4 (10.8)
DMFT, mean±SD	D	2.7 ± 4.0	1.9 ± 3.0	2.9 ± 4.0	3.7 ± 4.6	2.9 ± 6.5	1.7 ± 3.0
	M	12.1 ± 9.4	12.4 ± 8.8	11.1 ± 9.6	12.1 ± 8.6	19.1 ± 11.2	13.4 ± 9.7
	F	6.9 ± 6.1	7.2 ± 6.1	7.4 ± 6.2	6.6 ± 6.2	2.9 ± 4.2	6.1 ± 5.5
	DMFT sum	21.7 ± 6.0	21.5 ± 6.1	21.4 ± 5.8	22.3 ± 5.6	24.9 ± 4.3	21.2 ± 7.6
Number of teeth per patient, mean±SD		15.9 ± 9.4	15.6 ± 8.8	16.9 ± 9.6	15.9 ± 8.6	8.9 ± 11.2	14.6 ± 9.7
Edentulous patients, n (%)		45 (12.1)	7 (8.1)	23 (13.2)	5 (8.5)	5 (33.3)	5 (13.5)
Number of teeth in dentulous patients, mean±SD *(n = 326)*		18.0 ± 7.8	16.9 ± 7.8	19.4 ± 7.4	17.4 ± 7.4	13.3 ± 11.3	16.8 ± 8.4
Root canal treated teeth in dentulous patients, mean±SD *(n = 316)*	all	1.2 ± 1.7	1.2 ± 1.9	1.2 ± 1.6	1.5 ± 2.1	0.7 ± 1.3	1.1 ± 1.4
	without periodontitis apicalis	0.6 ± 1.2	0.6 ± 1.2	0.6 ± 1.2	0.7 ± 1.5	0.1 ± 0.3	0.6 ± 1.1
	with periodontitis apicalis	0.5 ± 0.9	0.5 ± 1.0	0.4 ± 0.8	0.6 ± 1.1	0.6 ± 1.3	0.4 ± 0.7
Number of teeth with periodontitis apicalis but without root canal treatment, mean±SD *(n = 316)*		0.7 ± 1.3	0.7 ± 1.4	0.8 ± 1.4	0.6 ± 1.0	1.5 ± 2.0	0.6 ± 1.3
Averaged radiographic bone loss in dentulous patients, mean±SD *(n = 316)*	mm	3.8 ± 1.7	4.0 ± 1.6	3.8 ± 1.8	3.8 ± 1.5	4.3 ± 2.5	3.6 ± 1.1
	%	13.2 ± 9.9	14.2 ± 9.6	12.9 ± 10.4	13.1 ± 9.7	15.9 ± 15.5	11.3 ± 6.3
	% / age	0.2 ± 0.2	0.2 ± 0.2	0.2 ± 0.2	0.2 ± 0.2	0.2 ± 0.2	0.2 ± 0.1

Regarding the oral health status, no significant differences between different anatomical site were found (p_adj._>0.05). For some variables, the number of patients is reduced due to missing data/radiographs.

^a^Note that oropharyngeal squamous cell carcinom (OPSCC) patients with known HPV status (n = 119) were analysed in a previous study on potential differences between HPV-positive and HPV-negative OPSCC patients [17].

**Fig 1:**
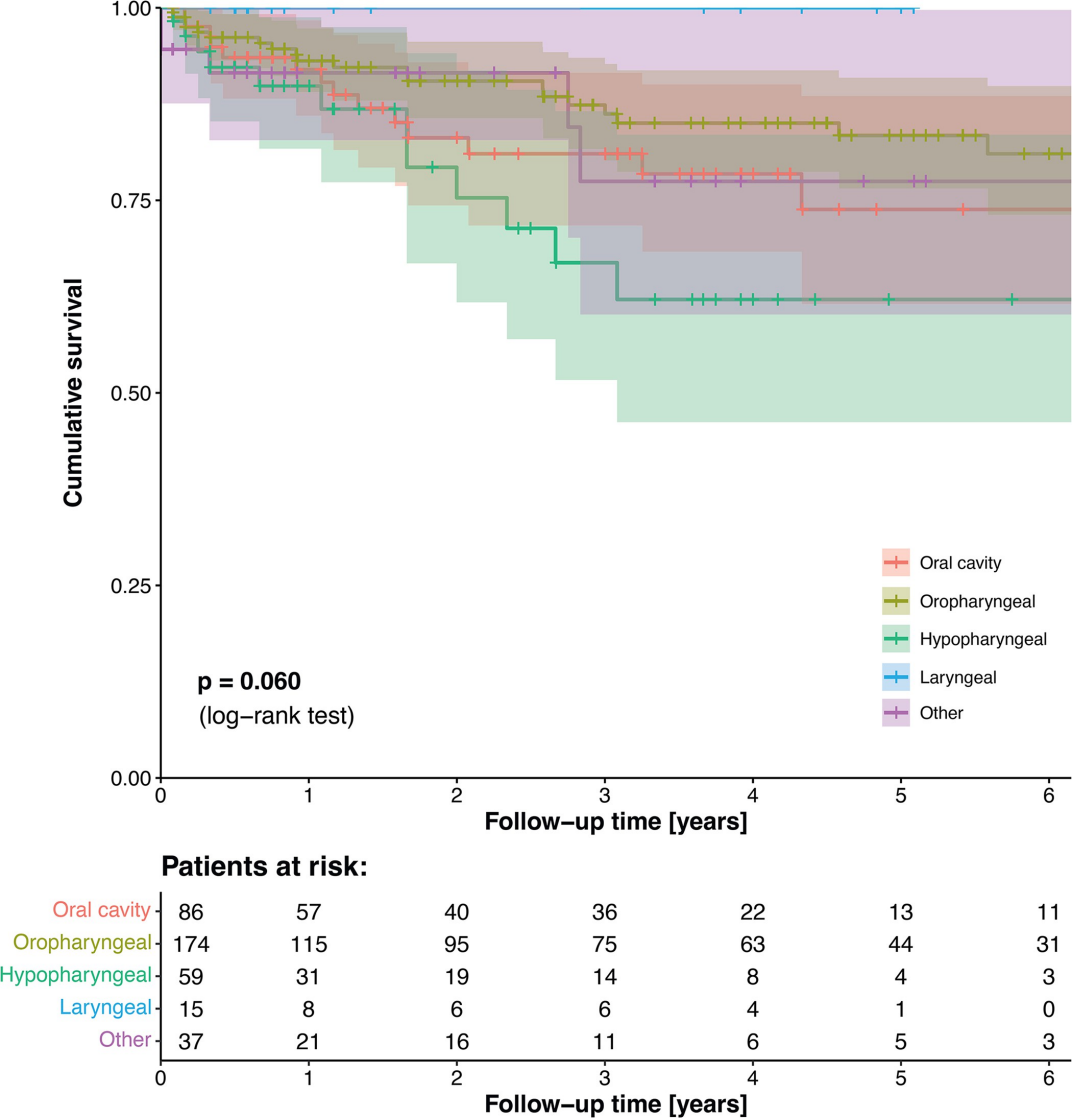
Separate Kaplan-Meier survival plots for each anatomical site.

Overall, five-year cumulative survival amounted to 78.6% (95%-CI: 73.1–84.5%). Survival probability did not vary between subsets of different anatomical sites (p = 0.060, Fig 1).

Subsequently, variables associated with the survival of all HNSCC patients and separately for each anatomical site are presented. Across all HNSCC patients, survival was associated with the anatomical site (p = 0.039), tobacco exposure (p = 0.091), alcohol consumption (p = 0.043), HPV status (p = 0.011), primary treatment (p = 0.079), treatment intent (p<0.001), performance status (p<0.001), and the number of teeth in dentulous patients (p = 0.071) in a univariable analysis (Table 2). However, none of these variables remained significant in the multivariable analysis.

**Table 2:** Univariable effect of patient-related risk factors and oral health parameters on survival for all HNSCC patients and each subset.

			Total	Oral	Oropharyngeal	Hypopharyngeal	Laryngeal	Other
Age at diagnosis			0.718	0.208	0.231	0.064 *	0.168	0.014 **
Anatomical site			0.039 **	–	–	–	–	–
Sex, male vs. female			0.746	0.174	0.724	0.614	N/A	0.602
Tobacco exposure, former/current smoker vs. never smoker			0.091 *	0.869	0.112	0.067 *	0.741	0.805
Alcohol consumption, former/current vs. never			0.043 **	0.223	0.209	0.095 *	0.373	0.146
HPV status (HPV-positive vs. HPV-negative)			0.011 **	0.407	0.010 **	0.969	0.487	0.096 *
Primary treatment (surgery +/- adj. radiotherapy vs. radiotherapy / radiochemotherapy)			0.079 *	0.722	0.148	0.374	0.250	0.613
Treatment intent (curative vs. palliative)			<0.001 **	0.007 **	0.006 **	0.227	0.322	N/A
Performance status			<0.001 **	0.206	<0.001 **	0.722	0.491	0.017 **
T stage			0.238	0.872	0.032 **	0.516	0.137	0.659
N stage			0.123	0.849	0.003 **	0.856	0.918	0.585
M stage (M1 vs. M0)			0.320	0.550	0.596	0.817	0.467	0.364
DMFT	D		0.758	0.651	0.222	0.612	0.110	0.340
	M		0.190	0.235	0.029 **	0.877	0.228	0.294
	F		0.373	0.177	0.003 **	0.908	0.787	0.812
	DMFT sum		0.198	0.645	0.161	0.956	0.407	0.232
Number of teeth per patient			0.190	0.235	0.029 **	0.877	0.228	0.294
Edentoulus patients			0.753	0.174	0.868	0.856	0.689	0.571
Number of teeth in dentulous patients			0.071 *	0.429	0.006 **	0.953	0.239	0.445
Root canal treated teeth in dentulous patients		all	0.385	0.048 **	0.502	0.697	0.843	0.870
		without periodontitis apicalis	0.246	0.056 *	0.749	0.534	0.163	0.697
		with periodontitis apicalis	0.415	0.219	0.259	0.766	0.995	0.823
Number of teeth with periodontitis apicalis but without root canal treatment			0.348	0.660	0.202	0.634	0.103	0.394
Averaged radiographic bone loss in dentulous patients		mm	0.975	0.104	0.255	0.498	0.707	0.379
		%	0.968	0.170	0.250	0.700	0.748	0.315
		% / age	0.795	0.175	0.468	0.541	0.664	0.137

P-values from likelihood ratio tests against the null model (* p<0.1, ** p<0.05).

Separately for the individual anatomical sites (oral cavity, oropharynx, hypopharynx, and other), patients’ survival was associated with some of the assessed variables: In patients with HNSCC originating in the oral cavity, survival was associated with the treatment intent (p = 0.007) and the number of root canal treated teeth in dentulous patients (all teeth: p = 0.048, teeth without periodontitis apicalis: p = 0.056). In patients with HNSCC originating in the oropharynx, survival was associated with the HPV status (p = 0.010), treatment intent (p = 0.006), performance status (p<0.001), T sage (p = 0.032), N stage (p = 0.003), the number of missing and filled teeth (p = 0.029 and p = 0.003, respectively), the number of teeth per patient (among all patients: p = 0.029, among dentulous patients: p = 0.006). In patients with HNSCC originating in the hypopharynx, survival was associated with the age at diagnosis (p = 0.064), tobacco exposure (p = 0.067), and alcohol consumption (p = 0.095). In patients with HNSCC originating in other sites, survival was associated with the age at diagnosis (p = 0.014), HPV status (p = 0.096), and performance status (p = 0.017). In patients with HNSCC originating in the larynx, none of the assessed variables were associated with survival. Further variables (e.g. sex) did not impact survival of all HNSCC patients and within each subset (see Table 2 for details).

Only for HNSCC originating in the oral cavity and in the oropharynx, significant risk factors were identified in the respective multivariable analyses: For HNSCC originating in the oral cavity, a palliative treatment intent remained a significant risk factor in the multivariable analysis (p = 0.015, HR = 4.54, Table 3). For HNSCC originating in the oropharynx, a low performance status was shown to be a significant risk factor in the multivariable analysis (p = 0.007, HR = 1.11, Table 4). No predictors remained significant in the respective multivariable analyses of the remaining subsets.

**Table 3:** Multivariable cox regression analysis for HNSCC originating in the oral cavity including risk factors with p-values ≤0.10 in the univariable analysis.

		Hazard Ratio	95%-CI	P-value
Palliative treatment intent [Ref.: curative]		4.54	1.34–15.38	0.015 *
Root canal treated teeth in dentulous patients	all			0.759
	without periodontitis apicalis			0.600

Likelihood ratio test against the null model: p = 0.030 (* p<0.05).

**Table 4:** Multivariable cox regression analysis for HNSCC originating in the oropharynx including risk factors with p-values ≤0.10 in the univariable analysis.

		Hazard Ratio	95%-CI	P-value
HPV-positive [Ref.: HPV-negative]				0.261
Palliative treatment intent [Ref.: curative]				0.749
Performance status		0.90	0.84–0.97	0.007 *
T stage				0.518
N stage				0.123
DMFT	M			0.306
	F			0.325
Number of teeth in dentulous patients				N/A

The number of teeth in dentulous patients was not entered as an independent variable due to multicollinearity. Likelihood ratio test against the null model: p = 0.003 (* p<0.05).

## Discussion

This study has found no differences in oral health parameters between different subsets of HNSCC patients and no significant effect of oral health parameters on patients’ survival. Only a palliative treatment intent and a low performance status were found to be independent risk factors for the survival of patients with HNSCC originating in the oral cavity or oropharynx, respectively. Thus, our results fail to reject the null hypothesis.

With regard to oral health in different subsets of HNSCC patients, the results of the present study are in line with two previous studies, where the DMFT varied between 15 and 21 [1] or 16.2 and 18.3 [2], the number of edentulous patients varied between 0 and 37% [1], and the number of present teeth varied between 17.2 and 21.1 [2]. Due to different assessment criteria, the periodontal status cannot be directly compared across these studies.

No significant differences between the different subsets of HNSCC patients with regard to oral health parameters were found, potentially as the groups were quite homogenous especially with regard to alcohol and tobacco exposure, which are not only the primary risk factors for HNSCC, but also relevant risk factors for oral diseases [18–21]. Besides shared risk factors, a direct link between periodontitis and cancer risk is likely, for example due to DNA damage or mutation caused by free radicals, cytokines or chemokines produced during the inflammatory process of periodontitis [22].

Among HNSCC patients, patients with oropharyngeal squamous cell carcinoma (OPSCC) present a specific risk factor profile including the infection with high-risk genotypes of the human papillomavirus (HPV). HPV-positive OPSCC patients tend to be younger, have lower alcohol intake and tobacco exposure, and show better therapeutical response resulting in superior overall survival compared to HPV-negative patients [23]. Therefore, OPSCC patients with known HPV status (119 of 174) were analysed in a previous study to detect potential differences in oral health parameters between HPV-positive and HPV-negative patients. Interestingly, HPV-positive OPSCC patients showed a better oral health, namely significantly more present teeth, a lower DMFT and were less often edentulous, compared to HPV-negative patients [17]. However, in line with the present study, no significant effect of oral health parameters on patients’ survival was detected in the multivariable analysis.

With regard of the survival of all HNSCC patients and of the patients of the different subsets, no oral health parameter remained significant in the multivariable analyses. Three previous studies found routine dental visits or good oral health care behavior to be associated with or to significantly impact survival of HNSCC patients [10–12]. However, all of these studies used self-reported proxy variables for oral health (e.g. frequency of dental exams, toothbrushing frequency, history of gum disease or tooth loss) rather than objective oral health parameters representing periodontal disease or caries experience. Good oral hygiene or good oral health might be surrogate parameters for a better general health or a healthier lifestyle contributing to a decreased mortality risk [12]. Farquhar et al. [12] and Tasoulas et al. [10] found an inverse effect of dental visit frequency on mortality risk that differed by cancer site: While Farquhar et al. [12] reported significantly lower hazard ratios for cancer of the oral cavity, laryngeal/hypopharyngeal sites, and the overall sample (all sites), Tasoulas et al. [10] only found significantly lower hazard ratios for oropharyngeal cancer and the overall sample. The effect of dental visits on survival was attributed to oral health, namely periodontitis, that might cause changes of the local environment (e.g. local inflammation, bacterial carcinogenesis) that might promote cancer development and behavior [12]. However, survival of HNSCC patients is heavily influenced by other factors such as patient prognosis and treatment.

This study analysed if oral health affects survival of HNSCC subsets differently. The main strength of this study is that objective clinical and radiological data rather than self-reported proxy variables for oral health were used. Data extraction and radiographic measurement were performed by a calibrated examiner. The examiner’s intra-rater reliability of the radiographic assessment and inter-rater reliability with a second examiner were shown to be excellent in a previous study on potential differences between HPV-positive and HPV-negative OPSCC patients [17], increasing the overall validity of our results. However, several limitations must be also addressed: Periodontal status was obtained only from radiographs as described previously [16], and no clinical periodontal variables (e.g. clinical attachment loss, inflammation status) were available. Therefore, the clinical periodontal status might vary (ie, being under or overestimated in our study). This aspect might explain the missing association between periodontal disease and patients’ survival. Moreover, a potential selection bias of the included patients must be discussed, as only patients prior to planned radio(chemo)therapy were included. Patients undergoing surgical therapy only were not included as they are usually not referred to the standard dental assessment [24]. Thus, a comparatively small subset of patients with laryngeal squamous cell carcinoma was examined and the underlying analysis might be underpowered, as these patients often undergo surgical treatment only. Also, follow-up was censored at patients’ last known visit to our hospital. Due to subsequent outpatient (dental) treatment, this study has a comparatively short follow-up time. Consequently, oral health and its effect on survival in the HNSCC subsets might be different from the present cohort, if also patients with early stages of HNSCC or indicated for surgical therapy were included.

## Conclusions

Oral health was not different between different subsets and had no significant effect on survival of HNSCC patients.

## Supporting information

S1 File Dataset. (CSV)
